# Shear Wave Elastography of the Abductor Pollicis Brevis in Carpal Tunnel Syndrome: Relationship with Quantitative Motor Unit Potential and Nerve Conduction Parameters

**DOI:** 10.3390/jcm15197507

**Published:** 2026-09-27

**Authors:** Hasan Kara, İlknur Albayrak Gezer, İsa Cüce, Servin Karaca, Fatma Nur Aba, Hakan İmamoğlu

**Affiliations:** 1 Department of Physical Medicine and Rehabilitation, Erciyes University Faculty of Medicine, 38039 Kayseri, Türkiye; icuce@erciyes.edu.tr (İ.C.); sarvinmoloudnejad72@gmail.com (S.K.); 2 Department of Physical Medicine and Rehabilitation, Selçuk University Faculty of Medicine, 42130 Konya, Türkiye; ilknur.ftr@gmail.com; 3 Department of Physical Medicine and Rehabilitation, Nevşehir State Hospital, 50300 Nevşehir, Türkiye; fatmanuraslan05@gmail.com; 4 Department of Radiology, Erciyes University Faculty of Medicine, 38039 Kayseri, Türkiye; himamoglu@erciyes.edu.tr

**Keywords:** carpal tunnel syndrome, shear wave elastography, abductor pollicis brevis, motor unit potential, needle electromyography, nerve conduction studies

## Abstract

**Background/Objectives:** We examined the association between shear wave elastography (SWE) of the abductor pollicis brevis (APB) and quantitative motor unit potential (MUP) parameters in carpal tunnel syndrome (CTS), using the ulnar-innervated abductor digiti minimi (ADM) as a reference muscle. **Methods:** In this prospective cross-sectional study, 73 patients with electrodiagnostically confirmed CTS (125 hands) and 37 healthy controls (69 hands) underwent SWE of the median nerve, APB, and ADM. Quantitative MUP analysis of the APB was performed in 65 hands with moderate or severe CTS. Both hands were included and analysed using mixed-effects models with participant as a random intercept; correlations were corrected using the Benjamini–Hochberg procedure. **Results:** The median nerve cross-sectional area, Young’s modulus, and shear wave velocity were higher in CTS hands than in controls (all *p* < 0.001), as were APB Young’s modulus (difference 2.85 kPa, 95% CI 0.46 to 5.24; *p* = 0.020) and shear wave velocity (*p* = 0.027). The ADM measurements and the within-hand APB/ADM ratio did not differ significantly. APB stiffness did not correlate with any MUP or motor conduction parameter. Weak correlations with sensory distal latency and sensory conduction velocity did not survive correction for multiple comparisons. **Conclusions:** Increased stiffness of the median nerve and the APB in CTS was not accompanied by a difference in the ulnar-innervated ADM, but the within-hand APB/ADM ratio did not differ either, so anatomical specificity was not established. Increased APB stiffness represented a group-level mechanical change that, in moderate-to-severe CTS, did not track neurogenic motor unit remodelling as measured by needle electromyography, and its clinical role remains to be defined.

## 1. Introduction

Carpal tunnel syndrome (CTS) results from compression of the median nerve within the carpal tunnel, and its pathophysiology follows a characteristic progression: sensory fibres are typically affected first, and continued compression subsequently involves motor fibres, leading to weakness and denervation of the median-innervated thenar muscles, particularly the abductor pollicis brevis (APB) [1,2,3,4,5]. In the absence of timely intervention, this process can progress to irreversible muscle atrophy [6]. Early and accurate identification of muscle involvement is therefore of considerable clinical importance for guiding treatment decisions and preventing permanent functional loss.

Electrodiagnostic testing is widely regarded as the reference standard for confirming the diagnosis of CTS and for assessing the degree of nerve and muscle involvement [4,7,8,9]. Needle electromyography (EMG), in particular, provides direct evidence of muscle involvement: spontaneous activity indicates active denervation, whereas changes in motor unit potentials reflect the reinnervation that follows it [10,11]. Despite its diagnostic value, needle EMG is an invasive procedure associated with discomfort and, occasionally, pain, and its diagnostic accuracy depends on adequate patient cooperation [12,13]. These limitations have motivated growing interest in non-invasive imaging alternatives capable of detecting muscle changes associated with CTS [14].

Ultrasonography, specifically shear wave elastography (SWE), has been proposed as a non-invasive method for quantifying tissue stiffness in real time [15,16,17]. Median nerve stiffness has consistently been found to be elevated in patients with CTS compared with healthy controls [18,19,20]. More recently, SWE evaluation has been extended to the thenar musculature, with discordant results. Shin et al. found thenar shear wave velocity to be higher in CTS wrists than in controls, yet to correlate inversely with electrodiagnostic severity [21]. Grönfors et al., in the only study to have combined thenar SWE with needle EMG, reported the opposite direction: velocity increased with neurophysiological severity and was faster in muscles showing neurogenic findings [22]. In that study, however, neurogenic change was recorded dichotomously as present or absent, and only seven muscles met the definition. A binary classification separates muscles with neurogenic changes from those without, but it cannot express how extensively the surviving motor units have been reorganised. Quantitative motor unit potential (MUP) parameters (duration, amplitude, number of phases, and polyphasia) describe this reorganisation on a continuous scale, so that every muscle contributes its own value rather than only those crossing a categorical threshold, and the relationship with stiffness can be examined across the whole range of involvement. Whether muscle stiffness varies with the extent of this reorganisation has not been examined.

The primary aim of the present study was to examine the relationship between APB muscle stiffness assessed using SWE and the extent of neurogenic motor unit remodelling, quantified from MUP parameters rather than classified as present or absent. The secondary aim was to compare SWE measurements of the median nerve, APB muscle, and ulnar-innervated abductor digiti minimi (ADM) muscle between patients with CTS and healthy controls, using the ADM as a reference muscle to examine whether any observed changes were restricted to the median nerve territory.

## 2. Materials and Methods

### 2.1. Study Design, Setting, and Participants

This prospective cross-sectional study was conducted at the Department of Physical Medicine and Rehabilitation, Erciyes University Faculty of Medicine Hospital, between January 2024 and February 2026. The study was approved by the Erciyes University Clinical Research Ethics Committee (Approval No. 2023/273) and conducted in accordance with the Declaration of Helsinki. Written informed consent was obtained from all participants.

Consecutive patients referred to the electroneuromyography unit from outpatient clinics with a preliminary diagnosis of CTS were assessed for eligibility. Nerve conduction studies (NCS) were performed first, and patients in whom the findings were consistent with CTS were screened against the inclusion and exclusion criteria. Eligible patients then underwent ultrasonographic assessment. In hands classified as moderate or severe CTS based on nerve conduction findings, quantitative MUP analysis was subsequently performed using needle EMG. This order was fixed by design: all ultrasonographic measurements were completed before any needle was inserted so that local tissue changes caused by the needle examination could not influence the elastographic values. Healthy volunteers without upper-limb symptoms were recruited from hospital staff, patients’ relatives, and volunteers outside the clinic to form the control group.

Inclusion criteria for the CTS group were age between 18 and 70 years, symptoms consistent with CTS, and electrodiagnostic confirmation of the diagnosis. Exclusion criteria were diabetes mellitus, polyneuropathy, rheumatic disease, thyroid disease, renal failure, pregnancy, a history of fracture or surgery of the affected extremity, systemic neurological disease, mass lesion, peripheral nerve disorders other than CTS, and cervical radiculopathy or neck pain persisting for at least three months. The exclusion criteria for the control group were acute or chronic musculoskeletal pain, any chronic disease, long-term medication use, and paraesthetic symptoms on clinical evaluation. Control participants were screened by a physician who was not involved in the ultrasound examinations. Screening comprised a structured symptom enquiry covering hand paraesthesia, nocturnal awakening, pain, and clumsiness, together with a hand examination that included inspection for thenar atrophy, sensory testing, manual muscle testing, and the Tinel and Phalen tests; volunteers with any positive finding were not enrolled. Ultrasound was not used to screen for subclinical median neuropathy, and no cross-sectional area threshold was applied when determining eligibility in either group. Controls were recruited consecutively with the intention of achieving broad similarity in age and sex distribution, but no formal individual or frequency matching was performed. Analyses were performed at the hand level. Hands with a bifid median nerve, an anatomical variant that precludes valid cross-sectional area and elastographic measurement, previous carpal tunnel surgery, or a history of trauma to the hand or wrist were excluded, as were unaffected hands of patients with unilateral disease (no symptoms and normal NCS). The bifid median nerve criterion was applied in both groups.

### 2.2. Electrodiagnostic Assessment

All patients underwent electrodiagnostic tests using a Natus Dantec Keypoint EMG system (software version 2.40; Natus Medical Inc., Middleton, WI, USA) in a temperature-controlled room maintained at 24–26 °C, with the hand temperature standardised for each participant. All electrodiagnostic studies were performed by a physiatrist with 9 years of experience in electrodiagnostic medicine, who was not involved in the ultrasonographic examinations. Median motor responses were recorded from the APB using the belly–tendon method, and median sensory responses were recorded orthodromically from the second digit to the wrist. Ulnar motor responses were recorded from the ADM using the belly–tendon method, and ulnar sensory responses were recorded orthodromically from the fifth digit to the wrist. Motor distal latency, motor conduction velocity, compound muscle action potential (CMAP) amplitude, sensory distal latency, sensory conduction velocity, and sensory nerve action potential (SNAP) amplitude were documented for each hand. CTS severity was classified at the hand level according to established electrodiagnostic criteria [23] as mild (slowing of median sensory conduction with normal motor distal latency), moderate (sensory slowing accompanied by prolonged motor distal latency), or severe (absent median sensory responses with prolonged motor distal latency). Control participants underwent only ultrasonographic assessment; no electrodiagnostic testing was performed in this group.

Needle EMG of the APB muscle was performed using a concentric needle electrode. MUPs were sampled from four quadrants of the muscle at four different depths, and a minimum of 20 distinct potentials were collected per muscle. Quantitative analysis was performed automatically using the device software (QEMG module), which computed the mean duration, amplitude, number of phases, and percentage of polyphasic potentials for each muscle. The physiatrist who performed and interpreted the needle EMG examination was blinded to the ultrasonographic findings. Spontaneous activity and interference patterns were evaluated as part of the routine examination but were not systematically recorded; therefore, they were not included in the analysis. Needle EMG was not performed on the ADM muscle or in hands with mild CTS, in which involvement is confined to sensory fibres and the motor distal latency is by definition normal, so that needle examination of the APB is not routinely indicated [10]. The correlation analyses involving MUP parameters were therefore restricted to hands with moderate or severe CTS.

### 2.3. Ultrasonographic Assessment

All ultrasonographic measurements were performed by a single physiatrist with over 10 years of experience in musculoskeletal ultrasonography under the supervision of a radiologist with expertise in musculoskeletal elastography. A GE LOGIQ P8 system (application software version R4, software revision 5.2; GE HealthCare Technologies, Inc., Chicago, IL, USA) with a 7–12 MHz linear-array transducer was used for all examinations. All measurements were obtained using the wrist preset of the system at its default frequency, with the imaging depth kept at 2 cm for the great majority of acquisitions; gain was adjusted for individual images and was not standardised. The examiner was blinded to the participants’ clinical findings and electrodiagnostic results.

The participants were examined while seated, facing the examiner, with the elbow flexed at 90°, the forearm supinated, and the wrist in a neutral position resting on a soft cushion. The fingers were held relaxed to avoid increasing carpal tunnel pressure, and no pressure was applied to the transducer. Before scanning, the wrist was allowed to rest on the cushion until the participant was relaxed, and after the transducer had been positioned, acquisition was delayed until the elastographic map had stabilised.

Grey-scale imaging was used to measure the cross-sectional area (CSA) of the median nerve, and SWE was used to measure the stiffness of the median nerve, APB, and ADM. The CSA of the median nerve was measured in the axial plane at the level of the proximal carpal tunnel by tracing a continuous outline of the inner border of the nerve. Circular regions of interest were used for all the elastographic measurements. The median nerve was imaged in the axial plane at the same level, with the region of interest positioned at the centre of the nerve [24]. The APB and ADM muscles were imaged with the transducer oriented longitudinally to the muscle fibres, as close as possible to the midpoint of the corresponding metacarpal bone and at the thickest part of the muscle belly; measurement sites were identified from anatomical landmarks on each occasion and were not marked on the skin [25]. The size of the acquisition box was adapted to the structure examined: for the median nerve it was set to encompass the whole nerve, and for the muscles it was extended to the superficial and deep fascial borders. The circular regions of interest were likewise adjusted rather than fixed, being made as large as the acquisition box allowed while excluding adjacent tendons and fascia [25]. Because the acquisition box and the region of interest were adapted to each structure, their dimensions and the stabilisation time were not recorded as fixed values.

For each structure, the system displayed two values: shear wave velocity in m/s and Young’s modulus in kPa (Figure 1). Neither was computed by the investigators. The kilopascal value is not an independent measurement but is derived by the system from the measured velocity (c) as E = 3ρc^2^, where ρ is the assumed tissue density of 1000 kg/m^3^ [15]; the paired values displayed throughout this study were consistent with this relationship. The two outputs therefore express the same underlying measurement in different units rather than providing independent information. A colour-coded reliability map was displayed alongside the stiffness map for every acquisition, but the system does not provide a numerical reliability index in this preset. The transducer orientation and position were therefore adjusted during scanning until this map was as uniform and as close to the most reliable end of the colour scale as could be achieved; approximately ten elastographic frames were then recorded for each structure, and the three frames with the most uniform and highest-quality reliability map were used for measurement. The mean of these three values was taken as the value for that structure.

### 2.4. Statistical Analysis

Statistical analyses were performed using IBM SPSS Statistics version 26.0 (IBM Corp., Armonk, NY, USA) and jamovi version 2.6.26 (The jamovi project, Sydney, Australia). The distribution of continuous variables was assessed separately in each group using the Shapiro–Wilk test. Continuous variables were presented as mean ± standard deviation; the median and interquartile range (IQR; 25th–75th percentile) are additionally given for the electrophysiological, motor unit potential and ultrasonographic variables. Because both hands were included for most participants, the observations were not independent. Comparisons between the CTS and control groups were therefore performed using linear mixed-effects models with group as a fixed effect and participant as a random intercept, estimated by restricted maximum likelihood with Satterthwaite approximation of the degrees of freedom. Results are reported as estimated mean differences with 95% confidence intervals. Participant-level continuous characteristics were compared using the independent-samples t test. Categorical variables were compared using the chi-square test or Fisher’s exact test when the expected cell counts were below five.

For the between-group comparisons both elastographic outputs are reported, for comparability with studies that have used either unit; the correlation analyses, however, were confined to a single metric, since the two outputs express the same measurement. The kilopascal value was selected for this purpose because muscle and nerve stiffness are conventionally expressed in this unit; as the two outputs are deterministically related, rank-based coefficients are identical for either choice. Correlations between the SWE parameters and both quantitative MUP and NCS parameters were examined using linear mixed-effects models fitted to rank-transformed, standardised variables, again with participant as a random intercept; the standardised regression coefficient of such a model is interpretable as a rank correlation coefficient that accounts for the non-independence of the two hands. In addition, the ratio of APB to ADM Young’s modulus was calculated within each hand as a within-hand comparator and was compared between the groups using the same mixed-effects model specification. Because no correction for multiple testing had been planned in advance, all correlation analyses were considered exploratory, and the *p*-values for the 20 correlations reported in Tables 3 and 4 were adjusted using the Benjamini–Hochberg false discovery rate procedure; the adjusted values are reported as q-values. Statistical significance was set at *p* < 0.05.

Intra-observer measurement reliability was assessed by re-measuring the archived images of 20 randomly selected APB acquisitions on a separate occasion, by the same examiner, blinded to the original values. Agreement was expressed as the intraclass correlation coefficient (ICC; two-way random, absolute agreement, single measures), the standard error of measurement (SEM), the coefficient of variation, and the minimal detectable change (MDC_95_ = 2.77 × SEM).

## 3. Results

A total of 80 patients with clinically suspected CTS and 50 healthy volunteers were assessed for eligibility. A total of 7 patients and 13 controls were excluded, leaving 73 patients and 37 controls for the analysis. At the hand level, 21 hands were excluded from the patient group (previous carpal tunnel surgery, *n* = 6; bifid median nerve, *n* = 1; hands with no symptoms and normal NCS, *n* = 14) and 5 hands from the control group (bifid median nerve, *n* = 3; history of trauma, *n* = 2), yielding 125 patient hands and 69 control hands for the analysis. The participant flow is shown in Figure 2.

The two groups did not differ significantly in age, sex distribution, body mass index, or hand dominance (Table 1). According to electrodiagnostic findings, 60 hands (48.0%) were classified as mild, 59 (47.2%) as moderate, and 6 (4.8%) as severe CTS.

Intra-observer measurement reliability for the APB was excellent (ICC 0.994, 95% CI 0.985 to 0.998; mean difference 0.25 kPa, *p* = 0.163), with a SEM of 0.54 kPa, a coefficient of variation of 2.4%, and an MDC_95_ of 1.49 kPa. The ultrasonographic findings are summarised in Table 2. The median nerve CSA, Young’s modulus, and shear wave velocity were all significantly higher in the CTS group than in the control group (all *p* < 0.001). Both APB Young’s modulus (*p* = 0.020) and shear wave velocity (*p* = 0.027) were also significantly increased in CTS hands. In contrast, neither ADM Young’s modulus (*p* = 0.452) nor shear wave velocity (*p* = 0.325) differed between the groups. The within-hand APB/ADM Young’s modulus ratio also did not differ between the groups.

None of the MUP parameters correlated significantly with the SWE measurements of either the APB muscle or the median nerve; all coefficients were weak (Table 3).

Before correction for multiple comparisons, APB stiffness correlated positively with sensory distal latency (ρ = 0.314, *p* = 0.003) and negatively with sensory conduction velocity (ρ = −0.262, *p* = 0.022), and median nerve stiffness correlated with sensory distal latency (ρ = 0.267, *p* = 0.049). No significant correlation was observed with any motor nerve conduction parameter, including CMAP amplitude. After correction, none of these associations remained statistically significant (all q ≥ 0.060) (Table 4).

## 4. Discussion

In this cross-sectional study, we examined whether APB stiffness measured by SWE reflects the extent of neurogenic motor unit remodelling quantified by MUP analysis in patients with CTS, and compared the elastographic measurements of the median nerve, APB, and ulnar-innervated ADM muscle with those of healthy controls. The median nerve CSA, Young’s modulus, and shear wave velocity were all significantly increased in CTS hands compared with controls, and APB stiffness was also higher, although the difference was modest. In contrast, no significant differences were observed in the ADM muscle or in the within-hand APB/ADM ratio. APB stiffness showed no significant correlation with any quantitative MUP parameter; weak correlations with sensory, but not motor, nerve conduction measures were observed before correction for multiple comparisons but did not remain significant afterwards.

In the present cohort, the median nerve CSA, Young’s modulus, and shear wave velocity were all higher in CTS hands than in controls. The enlargement in CSA reproduces a well-established sonographic feature of CTS and has been shown to correlate with electrodiagnostic severity [26,27,28]. The increase in stiffness is consistent with a systematic review of 17 studies comprising 1401 wrists, in which median nerve stiffness was elevated irrespective of the elastography mode used [18], and is generally attributed to the oedema, ischaemia, and fibrosis that follow sustained compression within the carpal tunnel [5]. Consistent with a reversible compressive component, median nerve stiffness and cross-sectional area have been shown to decrease six weeks after ultrasound-guided steroid injection [29]. Despite this apparent sensitivity to the compressive process, median nerve stiffness in our cohort was not significantly correlated with any nerve conduction or MUP parameter after correction for multiple comparisons. This appears to diverge from a body of work relating median nerve stiffness to the neurophysiological severity of CTS [19,20,21]. These studies, however, have related stiffness to ordinal severity categories, whereas we examined correlations with the continuous measures (distal latency, conduction velocity, and amplitude) from which such categories are constructed. These two approaches are not equivalent. Ordinal grading contrasts groups at opposite ends of the spectrum, whereas correlation across a narrow range within a diagnosed cohort is a more demanding test. Therefore, the associations reported in those studies may reflect a group-level difference rather than a graded relationship with any single measure.

In our cohort, APB stiffness was higher in CTS hands than in controls. This is consistent with Shin et al., who reported higher thenar muscle shear wave velocity in CTS wrists than in controls [21]. The values obtained in their patient group were similar to ours, which is notable given that both studies used a scanner from the same manufacturer and the same transducer orientation. However, our results differ from those of Tezcan et al., who reported lower APB stiffness in wrists with CTS among patients with psoriatic arthritis [30]. Their measurements were obtained in the horizontal plane rather than along the muscle fibres, and probe orientation is a recognised determinant of the values obtained in mechanically anisotropic tissue, such as skeletal muscle [25]. Their cohort also differed, comprising patients with an inflammatory rheumatic disease in whom CTS was predominantly mild. One possible explanation is that denervated muscle is replaced sequentially by fibrous and then adipose tissue, which affects stiffness in opposite directions [31,32], so that the stage of the disease at which measurements are obtained may influence the direction of the observed change. Region-of-interest placement and imaging depth are further recognised sources of variability in musculoskeletal shear wave elastography, and values obtained with one manufacturer’s system should not be compared directly with those from another [33].

In contrast, in our cohort, neither the Young’s modulus nor shear wave velocity of the ADM differed between groups, as also reported by Tezcan et al., the only other study to have measured this muscle in CTS [30]. Because the ADM is ulnar-innervated and no patient showed electrophysiological evidence of ulnar neuropathy, its stability is consistent with changes restricted to the median nerve territory. This interpretation requires caution, however: when the two muscles were compared within the same hand, the APB/ADM ratio did not differ between the groups, so our data are compatible with anatomical specificity without demonstrating it. The ADM is therefore best regarded as a reference muscle rather than a true internal control, since the two muscles differ in architecture, loading, geometry, and baseline stiffness; in our control hands the ADM was considerably stiffer than the APB (28.23 vs. 21.23 kPa).

The only previous study to combine APB elastography with needle EMG was that of Grönfors et al., who found a faster shear wave velocity in muscles with neurogenic findings than in those without [22]. Neurogenic change was recorded dichotomously, and only seven muscles met that definition, leading the authors to judge the method as unsuitable for clinical use in detecting denervation. Therefore, we quantified MUPs rather than classifying them. Duration, amplitude, number of phases, and polyphasia describe the size and synchrony of the surviving motor units, and increase as denervated fibres are reinnervated by collateral sprouting, providing a graded index of the reorganisation that follows axonal loss, although their relationship with the number of lost axons is not necessarily linear. Because this reorganisation is accompanied by structural changes within the muscle, stiffness may be expected to increase in parallel. Contrary to our hypothesis, APB stiffness was not significantly associated with any of the MUP parameters. Two considerations may account for these findings. First, the two measures index different processes: MUP abnormality is cumulative and persistent, whereas stiffness reflects muscle composition at the moment of measurement, which is thought to change sequentially from oedema to fibrosis to adipose replacement. Thus, a muscle with extensive but remote reinnervation may be electrically abnormal yet mechanically unremarkable. Second, and relatedly, the relationship is unlikely to be monotonic, since fibrosis raises stiffness while adipose infiltration lowers it [31,32], and in a rabbit model the elastic modulus of denervated muscle was reduced at two weeks and increased at eight weeks, in parallel with progressive collagen deposition [34]. This sequence is a hypothesis rather than an observation in our data, since muscle thickness, echogenicity, and fat infiltration were not quantified and no longitudinal imaging was performed. The literature is correspondingly discordant: thenar stiffness has been reported to correlate negatively with severity [21] and positively with neurogenic change [22], whereas in our analysis it was unrelated to the quantitative MUP parameters. A limitation of our own design should be set against this: needle EMG was not performed in mild CTS, so the analysis was confined to the moderate-to-severe range. Within such a restricted range the spread of motor unit remodelling is narrow and correlation coefficients are attenuated, and the wide confidence intervals of our estimates are consistent with this. With 65 hands, a correlation of about 0.34 would have been required for detection at 80% power (a calculation that does not account for the clustering of hands within participants and is therefore optimistic); the absence of a significant correlation therefore indicates a failure to demonstrate an association under these conditions rather than the absence of one across the CTS spectrum.

APB stiffness showed weak associations with sensory but not motor nerve conduction measures, correlating with sensory distal latency (ρ = 0.314, *p* = 0.003) and sensory conduction velocity (ρ = −0.262, *p* = 0.022). Neither association remained significant after correction for multiple comparisons (q = 0.060 and 0.220), and both should therefore be regarded as exploratory. Among the motor parameters, CMAP amplitude is of particular interest, since it is the standard electrophysiological index of motor axon loss to the APB, and APB stiffness was not related to it. Grönfors et al. likewise found that APB shear wave velocity was unrelated to CMAP amplitude in their overall sample, although an association emerged among patients younger than 60 years [22]. Taken with the absence of any correlation with MUP parameters, this consistency across two different electrophysiological indices of motor involvement provides no evidence that APB stiffness in CTS is determined principally by motor axonal loss or by the motor unit remodelling that follows it, although weak associations cannot be excluded. Since sensory fibres are affected earliest and most consistently in CTS, our findings instead raise the possibility that APB stiffness tracks the severity of median nerve compression, although this requires confirmation.

The magnitude of the APB difference warrants explicit consideration. The adjusted difference of 2.85 kPa (95% CI 0.46 to 5.24) is modest, and the corresponding descriptive effect size is moderate (Cohen’s d ≈ 0.5). It nevertheless exceeds the intra-observer measurement error observed in this study, being approximately five times the standard error of measurement and almost twice the minimal detectable change, so the separation between groups is unlikely to reflect measurement (reading) error. At the same time, the distributions overlapped substantially: with an effect of this size, a randomly selected CTS hand would be stiffer than a randomly selected control hand in only about 64% of comparisons. Increased APB stiffness is therefore best understood as a group-level mechanical difference rather than a property that distinguishes individual hands. This study was not designed as a diagnostic accuracy study, and discrimination and threshold analyses were therefore not undertaken; our conclusions are confined to group-level differences.

This study has several limitations. Its cross-sectional design cannot establish the temporal sequence of nerve compression, denervation, and changes in muscle stiffness. Symptom duration was not incorporated into the present analysis, a relevant omission given that the tissue changes following denervation are time-dependent and that the interpretation offered above rests on this dependence. Only six hands were classified as severe CTS, so the advanced end of the disease spectrum was sparsely represented. Control participants did not undergo electrodiagnostic testing, so subclinical median neuropathy among them cannot be excluded. SWE measurements are influenced by device settings, transducer orientation, and the degree of muscle relaxation; standardised reference values are not yet available; and the absolute values reported here should therefore be interpreted with caution rather than treated as generalisable thresholds. The reliability map available on this system is colour-coded rather than numerical, so acquisitions were accepted and the three frames used for measurement were selected visually rather than against a predefined numerical threshold. Finally, reliability was assessed by re-measuring archived images rather than by repeating the acquisitions, so variability arising from transducer repositioning is not captured.

## 5. Conclusions

In conclusion, SWE showed increased stiffness of both the median nerve and the APB muscle in hands with CTS, whereas the ulnar-innervated ADM muscle did not differ from controls. This pattern is compatible with changes restricted to the median nerve territory, although the within-hand APB/ADM ratio did not differ between the groups and therefore does not establish anatomical specificity. APB stiffness did not, however, correlate significantly with quantitative MUP parameters in moderate-to-severe CTS. The observation that APB stiffness related to sensory rather than motor conduction measures did not survive correction for multiple comparisons and should be regarded as hypothesis-generating rather than as evidence for a specific mechanism. Taken together, these findings indicate that APB stiffness and quantitative MUP analysis capture different aspects of muscle involvement in CTS; whether SWE provides clinically useful information alongside needle EMG cannot be determined from the present design and would require studies conducted for that purpose. Longitudinal studies incorporating symptom duration, repeated measurements, and motor unit number estimation across a broader range of disease severity would help clarify how muscle stiffness evolves during denervation and reinnervation.

## Figures and Tables

**Figure 1:**
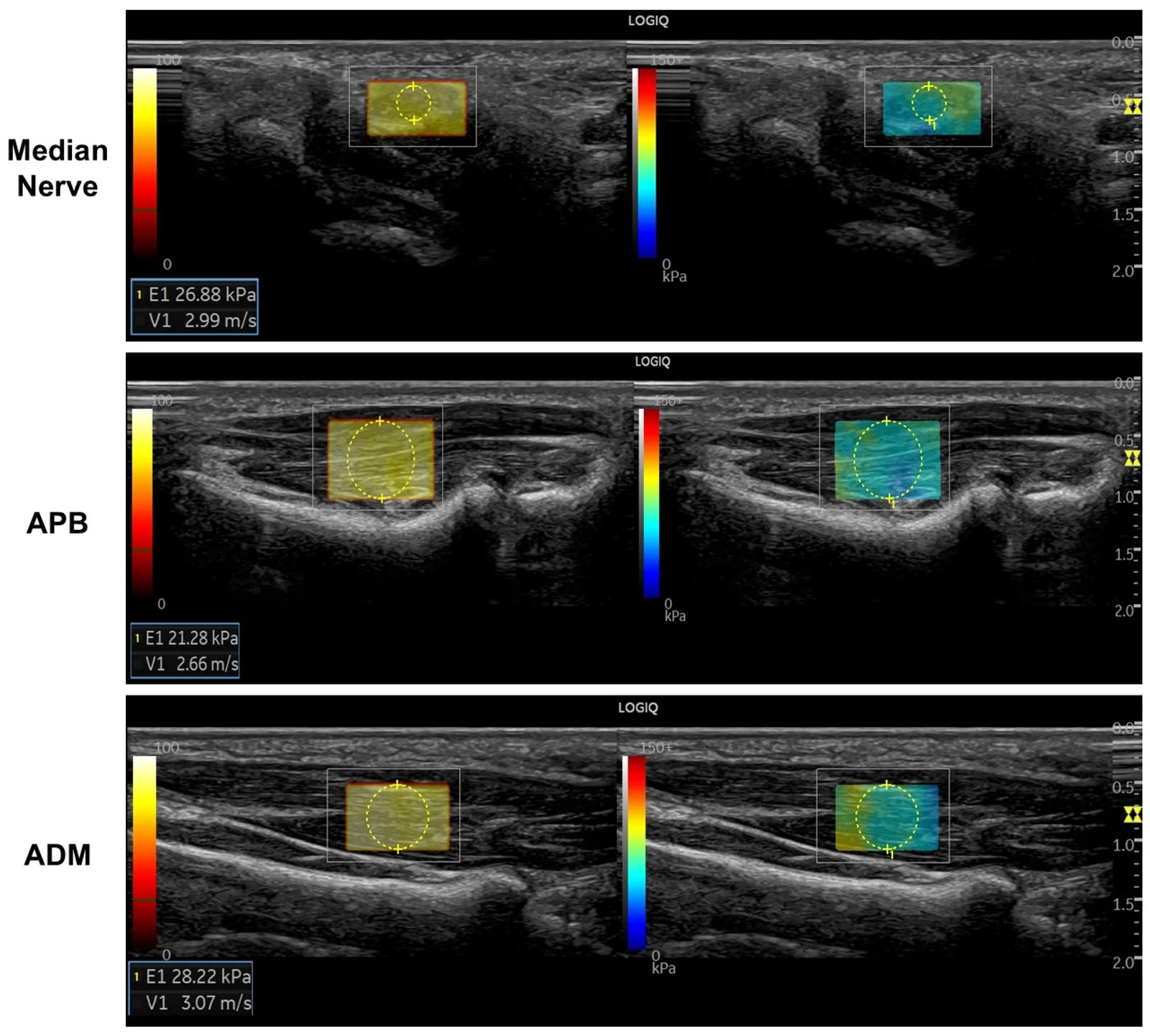
Representative shear wave elastography measurements of the median nerve, abductor pollicis brevis, and abductor digiti minimi. For each structure, the left panel shows the reliability measurement map and the right panel shows the stiffness map, with the circular region of interest indicated. The Young’s modulus (E, kPa) and shear wave velocity (V, m/s) values are displayed for each measurement. APB, abductor pollicis brevis; ADM, abductor digiti minimi.

**Figure 2:**
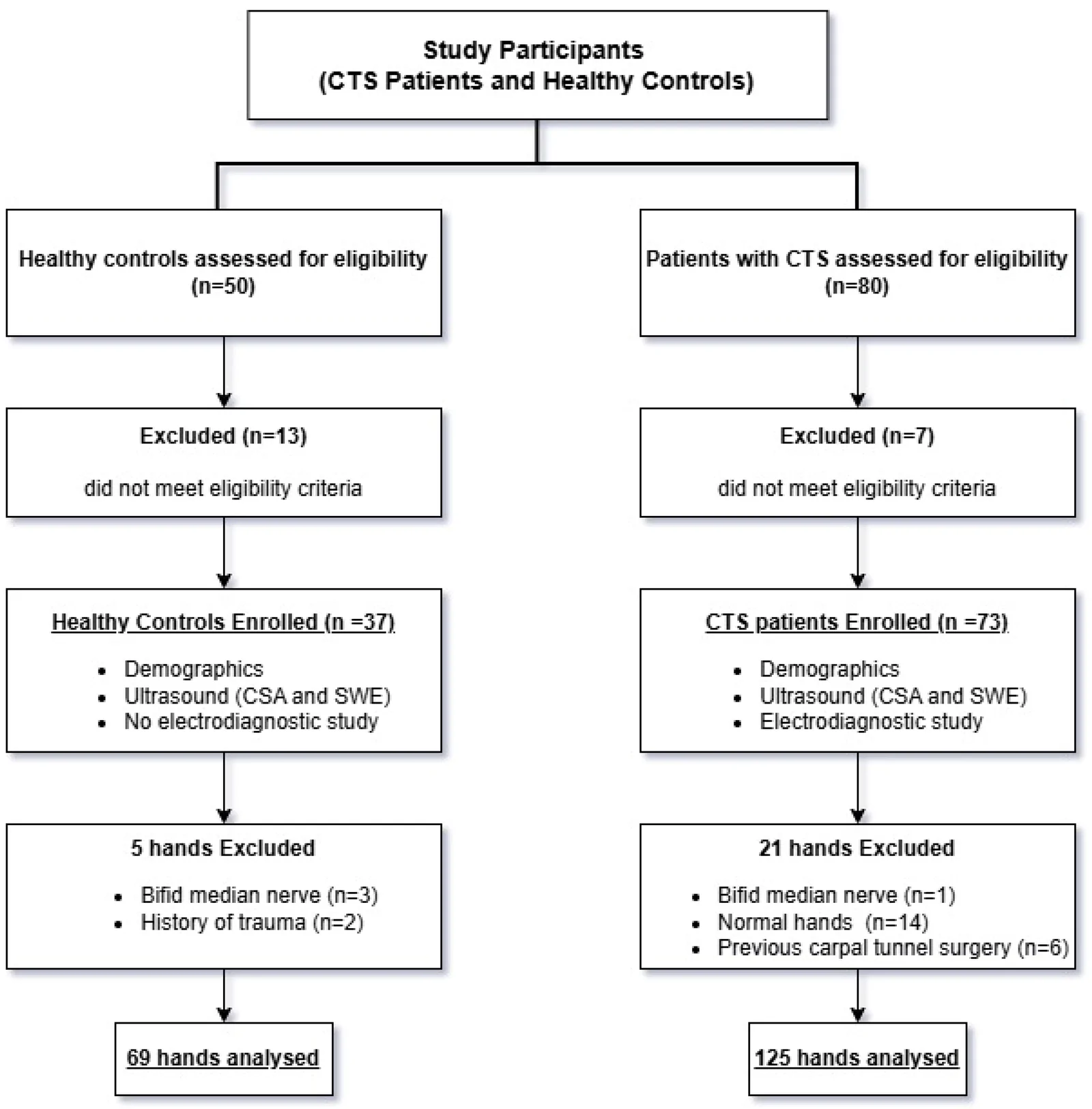
Flow of participants through the study. Analyses were performed at the hand level. “Normal hands” refers to the unaffected hands of patients with unilateral disease, defined as no symptoms together with normal nerve conduction studies. CTS, carpal tunnel syndrome; CSA, cross-sectional area; SWE, shear wave elastography.

**Table 1:** Demographic, Clinical, and Electrophysiological Characteristics of Participants.

Variable	Carpal Tunnel Syndrome (*n* = 73)	Healthy Controls (*n* = 37)	*p*-Value
**Age (years)**	51.67 ± 8.99	50.22 ± 10.01	0.442
**Sex, female:male, *n* (%)**	51:22 (69.9%:30.1%)	24:13 (64.9%:35.1%)	0.595
**BMI (kg/m^2^)**	30.23 ± 4.85	28.82 ± 3.79	0.125
**Dominant hand, right:left, *n* (%)**	68:5 (93.2%:6.8%)	31:6 (83.8%:16.2%)	0.177
**CTS severity (125 hands), *n* (%)**			
**• Mild**	60 (48.0%)	—	—
**• Moderate**	59 (47.2%)	—	—
**• Severe**	6 (4.8%)	—	—
**Motor nerve conduction (125 hands)**			
**• Distal latency (ms)**	4.01 ± 1.01 (3.69 [3.33–4.43])	—	—
**• Conduction velocity (m/s)**	54.40 ± 3.89 (54.10 [51.80–56.40])	—	—
**• CMAP amplitude (mV)**	13.40 ± 4.35 (13.20 [10.40–16.00])	—	—
**Sensory nerve conduction (119 hands)**			
**• Distal latency (ms)**	3.60 ± 0.65 (3.44 [3.16–3.94])	—	—
**• Conduction velocity (m/s)**	34.30 ± 5.66 (35.00 [30.57–38.38])	—	—
**• SNAP amplitude (µV)**	13.00 ± 7.18 (11.40 [7.28–16.90])	—	—
**Quantitative motor unit potential analysis (65 hands)**			
**• Duration (ms)**	10.70 ± 1.88 (11.10 [9.45–11.75])	—	—
**• Amplitude (µV)**	1310 ± 615 (1227 [975–1541])	—	—
**• Number of phases**	3.28 ± 0.32 (3.30 [3.05–3.50])	—	—
**• Polyphasia (%)**	13.10 ± 9.00 (12.00 [5.85–18.90])	—	—

Data are presented as mean ± SD unless otherwise indicated; electrophysiological values are presented as mean ± SD (median [interquartile range]). Demographic data refer to the participants, whereas CTS severity and electrophysiological findings are reported at the hand level; electrodiagnostic testing was performed only in patients, and needle EMG only in hands with moderate or severe CTS. CTS, carpal tunnel syndrome; BMI, body mass index; CMAP, compound muscle action potential; SNAP, sensory nerve action potential.

**Table 2:** Grey-Scale and Shear Wave Elastography Measurements of the Median Nerve, Abductor Pollicis Brevis, and Abductor Digiti Minimi.

	Carpal Tunnel Syndrome (125 Hands)		Healthy Controls (69 Hands)		Difference (95% CI)	*p*-Value
**Variable**	Mean ± SD	Median (IQR)	Mean ± SD	Median (IQR)		
**CSA of the median nerve (mm^2^)**	13.03 ± 3.66	12.52 (10.37–14.65)	8.63 ± 2.16	8.36 (7.29–9.43)	4.28 (2.79 to 5.77)	**<0.001**
**Median nerve, Young’s modulus (kPa)**	43.94 ± 16.27	38.34 (31.49–55.81)	26.70 ± 8.23	26.32 (22.16–30.70)	17.11 (10.62 to 23.60)	**<0.001**
**Median nerve SWV (m/s)**	3.76 ± 0.69	3.57 (3.24–4.31)	2.95 ± 0.46	2.96 (2.71–3.20)	0.81 (0.53 to 1.10)	**<0.001**
**APB, Young’s modulus (kPa)**	24.03 ± 6.46	23.00 (20.11–28.07)	21.23 ± 3.61	21.14 (19.87–23.27)	2.85 (0.46 to 5.24)	**0.020**
**APB SWV (m/s)**	2.80 ± 0.39	2.77 (2.59–3.06)	2.64 ± 0.24	2.66 (2.55–2.80)	0.16 (0.02 to 0.31)	**0.027**
**ADM, Young’s modulus (kPa)**	29.20 ± 6.53	29.09 (25.58–31.86)	28.23 ± 5.89	27.69 (24.91–30.43)	1.04 (−1.69 to 3.77)	0.452
**ADM SWV (m/s)**	3.10 ± 0.34	3.11 (2.92–3.26)	3.04 ± 0.34	3.03 (2.88–3.18)	0.07 (−0.07 to 0.21)	0.325
**APB/ADM Young’s modulus ratio**	0.84 ± 0.27	0.84 (0.65–1.02)	0.81 ± 0.24	0.77 (0.67–0.88)	0.03 (−0.08 to 0.15)	0.540

Data are presented as mean ± SD and as median (interquartile range, 25th–75th percentile) at the hand level. Between-group differences, 95% confidence intervals and *p*-values were estimated from linear mixed-effects models with group as a fixed effect and participant as a random intercept, accounting for the non-independence of the two hands of the same participant. The APB/ADM ratio was calculated within each hand from the Young’s modulus values (kPa), so that each hand served as its own comparator. Statistically significant values (*p* < 0.05) are shown in bold. CSA, cross-sectional area; SWV, shear wave velocity; APB, abductor pollicis brevis; ADM, abductor digiti minimi; CI, confidence interval.

**Table 3:** Correlation Between Shear Wave Elastography Parameters of the Abductor Pollicis Brevis Muscle and Median Nerve and Quantitative Motor Unit Potential Parameters in Patients with Moderate-to-Severe Carpal Tunnel Syndrome.

MUP Parameter	APB, Young’s Modulus (kPa)				Median Nerve, Young’s Modulus (kPa)			
	ρ	95% CI	*p*	q	ρ	95% CI	*p*	q
**Duration (ms)**	−0.081	−0.422 to 0.261	0.635	0.747	0.064	−0.403 to 0.532	0.778	0.778
**Amplitude (µV)**	−0.174	−0.499 to 0.151	0.284	0.568	−0.138	−0.587 to 0.311	0.530	0.707
**Number of phases**	−0.046	−0.376 to 0.284	0.778	0.778	0.257	−0.165 to 0.680	0.220	0.489
**Polyphasia (%)**	0.126	−0.200 to 0.453	0.438	0.674	0.102	−0.328 to 0.531	0.629	0.747

ρ, rank correlation coefficient estimated from linear mixed-effects models with participant as a random intercept; q, *p*-value adjusted for multiple comparisons (Benjamini–Hochberg) across the 20 correlations reported in Table 3 and Table 4. All correlation analyses were exploratory. APB, abductor pollicis brevis; MUP, motor unit potential; CI, confidence interval.

**Table 4:** Correlation Between Shear Wave Elastography Parameters of the Abductor Pollicis Brevis Muscle and Median Nerve and Median Nerve Conduction Study Parameters in Patients with Carpal Tunnel Syndrome.

NCS Parameter	APB, Young’s Modulus (kPa)				Median Nerve, Young’s Modulus (kPa)			
	ρ	95% CI	*p*	q	ρ	95% CI	*p*	q
*Motor Nerve Conduction Parameters*								
Distal latency (ms)	0.094	−0.121 to 0.309	0.390	0.650	0.240	−0.056 to 0.537	0.110	0.367
Conduction velocity (m/s)	−0.103	−0.320 to 0.115	0.352	0.640	−0.236	−0.511 to 0.039	0.091	0.364
CMAP amplitude (mV)	0.028	−0.169 to 0.226	0.776	0.778	−0.171	−0.419 to 0.078	0.174	0.435
*Sensory Nerve Conduction Parameters*								
Distal latency (ms)	0.314	0.106 to 0.521	0.003	0.060	0.267	0.001 to 0.534	0.049	0.270
Conduction velocity (m/s)	−0.262	−0.484 to −0.039	0.022	0.220	−0.272	−0.549 to 0.005	0.054	0.270
SNAP amplitude (µV)	−0.197	−0.475 to 0.082	0.163	0.435	−0.133	−0.561 to 0.294	0.530	0.707

ρ, rank correlation coefficient estimated from linear mixed-effects models with participant as a random intercept; q, *p*-value adjusted for multiple comparisons (Benjamini–Hochberg) across the 20 correlations reported in Table 3 and Table 4. All correlation analyses were exploratory. APB, abductor pollicis brevis; NCS, nerve conduction study; CMAP, compound muscle action potential; SNAP, sensory nerve action potential; CI, confidence interval.

## Data Availability

The data presented in this study are available on request from the corresponding author. The data are not publicly available due to privacy restrictions.
